# Pharmacokinetics of Eight Flavonoids in Rats Assayed by UPLC-MS/MS after Oral Administration of *Drynariae rhizoma* Extract

**DOI:** 10.1155/2018/4789196

**Published:** 2018-12-18

**Authors:** Zhan-Ling Xu, Ming-Yue Xu, Hai-Tao Wang, Qing-Xuan Xu, Ming-Yang Liu, Chun-Peng Jia, Fang Geng, Ning Zhang

**Affiliations:** ^1^Key Laboratory of Chinese Materia Medica, College of Pharmacy, College of Jiamusi, Heilongjiang University of Chinese Medicine, Harbin, Heilongjiang 150040, China; ^2^Key Laboratory of Photochemistry Biomaterials and Energy Storage Materials of Heilongjiang Province, College of Chemistry & Chemical Engineering, Harbin Normal University, Harbin 150025, China; ^3^Pharmacy Department, Harbin Hospital of Traditional Chinese Medicine, Harbin 150076, China; ^4^Crop Academy of Heilongjiang University, Harbin 150080, China

## Abstract

As a traditional Chinese medicine, *Drynariae rhizoma* (Kunze ex Mett.) J. Sm. has been used to treat osteoporosis and bone resorption for 2500 years. Based on the previous study and literature references, flavonoids were proved to be the most abundant and main active compounds of *Drynariae rhizoma* for osteoporosis treatment. In order to make good and rational use of *Drynariae rhizoma* in future, a rapid, sensitive, and selective ultraperformance liquid chromatography-mass spectrometry (UPLC-MS/MS) method was developed to investigate the pharmacokinetics of eight main flavonoids in rat plasma after oral administration of the *Drynariae rhizoma* extract, including neoeriocitrin, luteolin-7-*O*-*β*-*D*-glucoside, astragalin, naringin, eriodictyol, luteolin, naringenin, and kaempferol. Plasma samples' pretreatment involved a solid-phase extraction column. The separation was performed on an ACQUITY UPLC^TM^ BEH C_18_ column with a gradient mobile-phase system of acetonitrile and 1% acetic acid in water. The detection was performed using a triple quadrupole tandem mass spectrometer equipped with an electrospray ionization interface (ESI) by multiple reaction monitoring (MRM) in the positive ion mode. All calibration curves exhibited good linearity (*r* ^2^ > 0.9990) over the measured ranges. The intraday and interday precisions (RSD) were within 13.87%, and the accuracy (RE) ranged from −14.57% to −0.25% at three quality control levels. Extraction recovery, matrix effect, and stability were satisfactory. The pharmacokinetic characteristics of the eight flavonoids of interest were clearly elucidated.

## 1. Introduction

The traditional Chinese medicine *Drynariae rhizoma* (Kunze ex Mett.) J. Sm., commonly known as Gu-Sui-Bu, is a fern plant widely distributed in southern China. *Drynariae rhizoma* is effective for the treatment of osteoporosis and bone resorption [1]. *Drynariae rhizoma* contains various types of chemical constituents, including flavonoids, triterpenes, phenolic acids, and their glycosides. But flavonoids and their glycosides are the most abundant constituents of *Drynariae rhizoma* [2, 3]. Furthermore, flavonoids showed protective activities of osteoporosis, bone fractures, oxidative damage, and inflammation [4–10]. Total flavonoids in *Drynariae rhizoma* could activate the estrogen receptors and have the trend of replacing estrogen for clinical use [11, 12]. Flavonoids have been regarded as the principle constituents that contribute to the bioactivities of *Drynariae rhizoma.*

**Figure 1:**
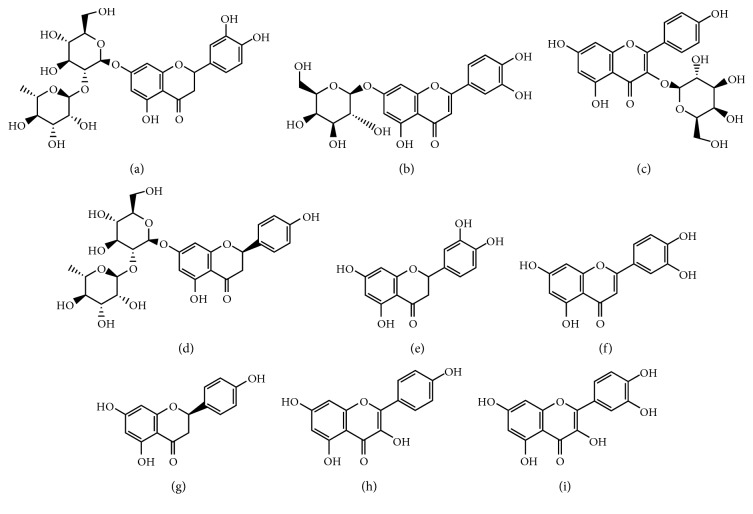
Chemical structures of eight analytes and IS. (a) Neoeriocitrin. (b) Luteolin-7-*O*-*β*-*D*-glucoside. (c) Astragalin. (d) Naringin. (e) Eriodictyol. (f) Luteolin. (g) Naringenin. (h) Kaempferol. (i) Quercetin.

In the bioactive research of *Drynariae rhizoma*, the key issue is how to study the effective substance of *Drynariae rhizoma* to play a key role in osteoporosis. Wang suggested a conceptual framework for illuminating the absorbed bioactive compounds in herb medicines [13]. In general, absorbed bioactive compounds more possibly play a part in the therapeutic effect *in vivo* after oral administration. Thus, it is necessary to measure the absorbed bioactive compounds in plasma to understand the effective substances in the herb. In our pilot study, eight flavonoids were detected in plasma after oral administration of *Drynariae rhizoma* extract (DRE), including neoeriocitrin, luteolin-7-*O*-*β*-*D*-glucoside, astragalin, naringin, eriodictyol, luteolin, naringenin, and kaempferol (Figure 1), which could contribute to the therapeutic effect of the DRE.

As far as we know, no study of the pharmacokinetic characteristics of the eight flavonoids in rats after oral administration of the DRE has been reported. Up to now, only one or two flavonoids or their metabolites of the DRE were determined in rat plasma, which could not fully reflect the drug metabolism process in the body after oral administration of DRE to humans or a model animal [14]. Therefore, it is necessary to establish an appropriate analysis method to characterize the pharmacokinetics of DRE *in vivo*.

In the present study, a rapid, sensitive, and selective UPLC-MS/MS method was developed and validated for the simultaneous determination of eight flavonoids from the DRE in rat plasma. Plasma samples were pretreated with the C_18_ SPE column. The detection was performed using a triple quadrupole tandem mass spectrometer equipped with an electrospray ionization interface (ESI) by multiple reaction monitoring (MRM) in the positive ion mode. The newly described UPLC-MS/MS method was validated and successfully applied to pharmacokinetic study of eight flavonoids in rats after oral administration of DRE.

## 2. Materials and Methods

### 2.1. Materials and Reagents

*Drynariae rhizoma* naturally grown in Jiangxi Province, China, was purchased from Anguo Herb Market (Hubei, China). Neoeriocitrin, luteolin-7-*O*-*β*-*D*-glucoside, astragalin, naringin, eriodictyol, luteolin, naringenin, and kaempferol and the internal standard (IS, quercetin) were purchased from Bailingwei Technology Co., Ltd. (Beijing, China) with purities >98%. Methanol and acetonitrile of the HPLC grade were obtained from Dikma (Richmond Hill, NY, USA). Acetic acid (HPLC grade) was purchased from Scharlau Chemie S. A. (Barcelona, Spain). Water was purified by redistillation and a Milli-Q® ultrapure water system (Millipore, Bedford, MA, USA). Other chemicals and solvents used were of analytical grade.

### 2.2. Instruments and Analytical Conditions

Liquid chromatography analysis was performed on an ACQUITY UPLC^TM^ system (Waters Corp., Milford, MA, USA), which included a cooling autosampler, column oven, and two pumps. The chromatographic separation was performed using an ACQUITY UPLC^TM^ BEH C_18_ column (2.1 × 50 mm, 1.7 *μ*m) at 35°C. The mobile phase consisted of 1% acetic acid in water as the aqueous phase (A) and 100% acetonitrile as the organic phase (B). The gradient elution program was 0–3 min, 10–30% B; 3–4.5 min, 30–40% B; 4.5–6 min, 40–70% B; and 6-7 min, 70–90% B. The flow rate was 0.3 ml/min, the temperature of the autosampler was 4°C, and the sample injection volume was 5 *μ*l.

Mass spectrometric detection was performed using a Waters® Micromass® Quattro Premier™ XE triple quadrupole tandem mass spectrometer equipped with an electrospray ionization interface in the positive ion mode. The ESI + source operation optimal parameters were capillary voltage 3.5 kV, source temperature 120°C, and desolvation temperature 300°C. Nitrogen was used as the desolvation and cone gas with flow rates of 600 and 30 L·h^−1^, respectively. Argon was used as the collision gas at a pressure of approximately 2.61 × 10^−3^ mbar. Quantitative analysis was performed in multiple reaction monitoring (MRM) mode, and the parent ion, daughter ion, cone voltage, and collision energies of eight analytes and the IS were optimized.  Table 1 shows product ion [M + H]^+^ MS spectra of the eight flavonoids of interest and the IS and their fragmentation pathways. All data collected in the centroid mode were processed using MassLynx™ NT 4.1 software with the QuanLynx™ program (Waters Corp.). The structure of the eight analytes are displayed in Figure 1.

### 2.3. Preparation of Standard and Quality Control Samples

Stock solutions of the standards for eight flavonoids and quercetin (IS) solution were prepared in a 10 ml volumetric flask. Each standard stock solution of 100 *μ*g/ml was prepared in methanol. The internal standard (quercetin) stock solution of 200 ng/ml was also dissolved with methanol. The plasma calibration standard solutions were prepared at concentrations in the range of 3.749–3749 ng/ml for neoeriocitrin, 1.856–4230 ng/ml for luteolin-7-*O*-*β*-*D*-glucoside, 1.317–1400 ng/ml for astragalin, 1.237–6370 ng/ml for naringin, 0.135–1040 ng/ml for eriodictyol, 2.742–3780 ng/ml for luteolin, 0.121–1210 ng/ml for naringenin, and 5.328–1209 ng/ml for kaempferol.

Three concentrations (low, middle, and high) of each analyte solution in drug-free plasma (40.4, 202, 2020 ng/ml for neoeriocitrin, 5.04, 50.4, 504 ng/ml for luteolin-7-*O*-*β*-*D*-glucoside, 5.1, 102, 1020 ng/ml for astragalin, 10.2, 408, 6120 ng/ml for naringin, 1.02, 40.8, 816 ng/ml for eriodictyol, 10.1, 40.4, 1010 ng/ml for luteolin, 5.2, 41.6, 208 ng/ml for naringenin, and 1.06, 42.4, 424 ng/ml for kaempferol) were used for quality control (QC) evaluation in UPLC-MS/MS analysis. All the solutions were stored at 4°C before use.

### 2.4. Preparation and Quality Assessment of DRE

The dried roots of *Drynariae rhizoma* (1 kg) were powdered and extracted three times under reflux in 15 times volume of methanol for every 2 hours. The solution was filtered and evaporated under reduced pressure in a Rotavapor R-3 rotary evaporator (Buchi Ltd., Labortechnik AG, Switzerland). Subsequently, the concentrated extract was dried in an oven, and the final weight of the DRE was 198.9 g with a yield of 19.9%. The contents of the main flavonoid constituents in DRE were quantitatively determined with the method described above, with the results of neoeriocitrin (5.1 mg/g), luteolin-7-*O-β*-*D*-glucoside (1.2 mg/g), astragalin (0.96 mg/g), naringin (8.5 mg/g), eriodictyol (0.66 mg/g), luteolin (0.11 mg/g), naringenin (2.71 mg/g), and kaempferol (0.14 mg/g), respectively.

### 2.5. Preparation of Plasma

Aliquots of plasma samples (400 *μ*l) were placed in a 10 ml centrifuge tube; 50 *μ*l of internal standard solution (200 ng/ml) and 50 *μ*l acetic acid were added. The mixture was vortexed for 1.0 min and then loaded onto an activated SPE C_18_ column (the SPE C_18_ column was activated by 3 ml of methanol before loading the sample, and the excess was washed off with 5 ml of purified water). After rinsing with 2 ml purified water, the SPE column was eluted with 4.5 ml of methanol and the eluate was evaporated to dryness under a gentle stream of nitrogen. The residue was reconstituted in 140 *μ*l methanol and centrifuged at 16000 × g for 15 min; then, a 5 *μ*l aliquot was injected into the UPLC-MS/MS system for analysis.

### 2.6. Method Validation

The method was validated in terms of specificity, linearity, lower limit of detection (LLOD), lower limit of quantification (LLOQ), accuracy and precision, extraction recovery, stability, and matrix effects based on the method validation procedure in the previous work [15].

#### 2.6.1. Specificity

We compared the chromatograms of blank plasma from six individual rats with those of corresponding plasma samples spiked with eight mixed standard samples and IS and plasma samples after oral administration of DRE.

#### 2.6.2. Linearity and Quantification

Calibration curves for the eight standards were constructed by plotting the peak area ratios of each analyte to that of the IS versus the corresponding concentration. The lower limit of detection (LLOD) was defined as the lowest concentration with a signal-to-noise ratio of 3 : 1. The lower limit of quantification (LLOQ) was determined as the lowest concentration of the calibration curve with a signal-to-noise ratio of 10 : 1.

#### 2.6.3. Precision and Accuracy

Intraday precision and interday precision and accuracy were assessed for low-, middle-, and high-concentration QC samples in six replicates on the same day and once a day for three consecutive days, respectively. Each tested sample was related to the calibration curve. The precision was calculated as the relative standard deviation (RSD%) and the accuracy as relative error (RE%). These results indicated that the precision and accuracy of the method were within acceptable limits. The intraday precision and interday precision and accuracy values for lowest acceptable reproducibility concentrations were denied as being within ±15%, and the precision and accuracy were within 80%–120%.

#### 2.6.4. Recovery and Matrix Effect

The recovery and matrix effects of analytes were determined for low-, middle-, and high-concentration QC samples with six replicates. Extraction recovery was determined at the three QC levels by comparing the peak areas of analytes between plasma samples spiked with analytes before and after extraction. Matrix effects were evaluated at the three QC levels by comparing the peak areas of analytes obtained from plasma samples spiked with analytes after extraction to those of pure standard solutions at the same concentrations; the acceptable range was 80–120%.

#### 2.6.5. Stability

The stability of determination was assessed by analysis of low-, middle-, and high-concentration QC samples with three replicates. The short-term stability was assessed by analyzing the QC samples kept in the autosampler (4°C) for 36 h. To assess long-term stability, the QC samples were stored at −20°C for 30 days. Freeze-thaw stability was evaluated by subjecting the QC plasma samples to three complete freeze/thaw cycles from −20°C to room temperature.

### 2.7. Pharmacokinetic Study of DRE

#### 2.7.1. Animals

Female Sprague Dawley rats (280–350 g) were supplied by the Animal Safety Evaluation Center of Heilongjiang University of Chinese Medicine (Harbin, Heilongjiang). All protocols for animal experiments were approved in accordance with the Regulations of Experimental Animal Administration issued by the State Commission of Science and Technology of the People's Republic of China. The rats were housed at 24 ± 2°C, and relative humidity was 60 ± 5%, with a 12 h-12 h light-dark cycle. Water and food were supplied freely.

#### 2.7.2. Drug Administration and Sampling

The UPLC-MS/MS method was successfully applied in a pharmacokinetic study of eight flavonoids in rats after oral administration of the DRE. The dosing solutions were freshly prepared DRE administrated via an oral gavage to the rats at a single dose of 4 g/kg. The animals had free access to water during the experiment. A series of blood samples were collected in 1.5 ml heparinized polythene tubes from the suborbital venous lexus of each rat at 0.08, 0.33, 0.5, 0.67, 1, 2, 4, 6, 8, 12, and 24 h after administration. The blood samples were centrifuged at 4500 × g for 10 min, and the supernatants were collected immediately and stored at −20°C until analysis.

#### 2.7.3. Data Analysis

Pharmacokinetic parameters were determined using a noncompartmental model and analyzed using pharmacokinetic software WinNonlin Standard Edition, version 1.1. Data are shown as the mean ± standard deviation (SD) for each parameter.

## 3. Results and Discussion

### 3.1. Optimization of LC-MS Conditions

#### 3.1.1. Optimization of Mass Spectrometric Conditions

To optimize the mass spectrometry conditions, appropriate mixed standard solutions of neoeriocitrin, luteolin-7-*O-β*-*D*-glucoside, astragalin, naringin, eriodictyol, luteolin, naringenin, kaempferol, and quercetin were monitored across a full scan in both positive and negative modes. The signal intensity and fragment stability in the positive mode were better than those in the negative mode for all the analytes. Therefore, the positive mode was used for analysis. The parent ion and daughter ion for each compound were obtained (Figure 1), and the cone voltage and collision energy for the eight analytes and the IS (quercetin) were optimized. The molecular weight, parent ion, daughter ion, cone voltage, collision energy, and retention time of the eight flavonoids and the IS are shown in Table 1.

#### 3.1.2. Chromatographic Conditions

To achieve symmetric peak shape and short running time for the simultaneous analysis of the eight analytes, we tested various mobile phase conditions to achieve good separation of the analytes. The mobile phase we finally optimized as 1% acetic acid-water as the aqueous phase (A) and 100% acetonitrile as the organic phase (B). In the optimized UPLC-MS/MS conditions for simultaneous determination of the eight compounds, all analytes were eluted rapidly with 5.0 min.

### 3.2. Method Validation

#### 3.2.1. Specificity

**Figure 2:**
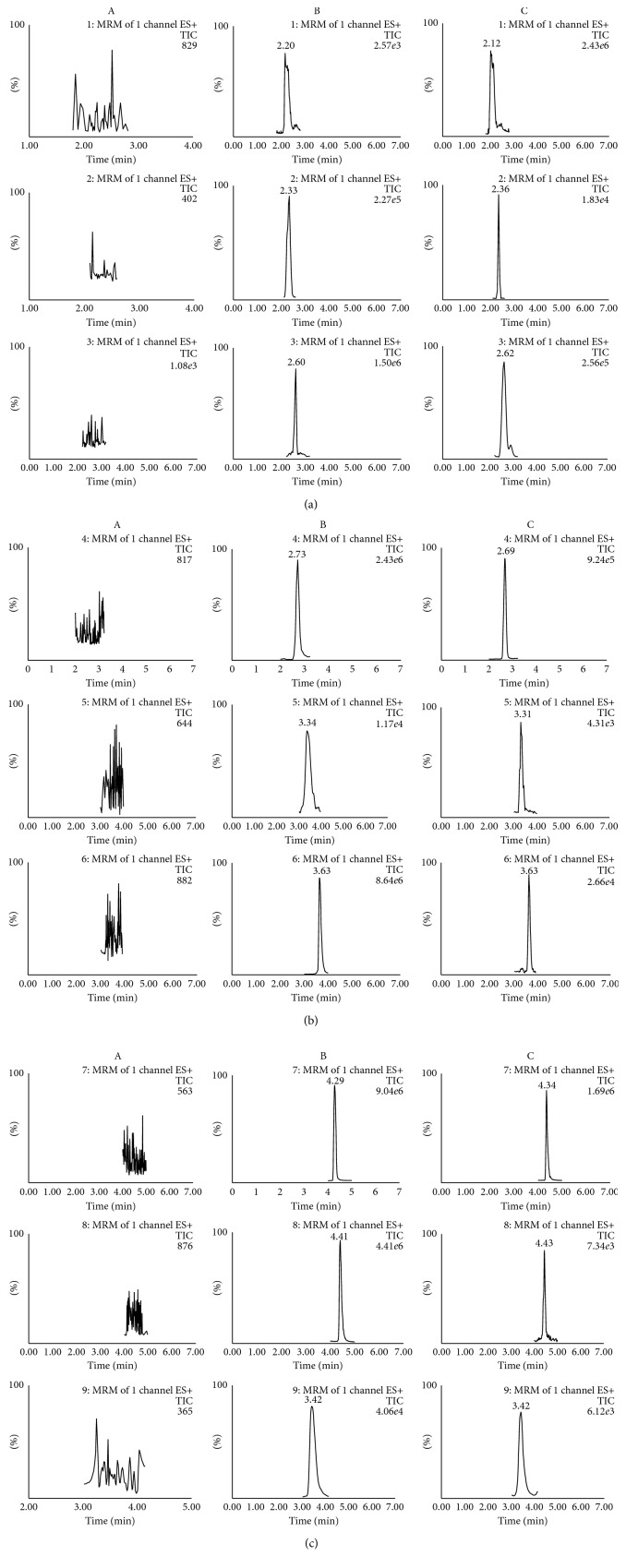
Representative MRM chromatograms of eight analytes and quercetin (IS). (a) Blank plasma. (b) Blank plasma spiked with the eight analytes and IS. (c) Plasma sample 1 h after oral administration of DRE (4 g/kg) (mean ± SD, *n*=6).

The retention times of neoeriocitrin, luteolin-7-*O*-*β*-*D*-glucoside, astragalin, naringin, eriodictyol, luteolin, naringenin, kaempferol, and IS were approximately 2.20 min, 2.36 min, 2.60 min, 2.69 min, 3.31 min, 3.63 min, 4.34 min, 4.43 min, and 3.42 min, respectively. The representative chromatograms of blank plasma, blank plasma spiked with reference standards and IS, and plasma obtained after the oral administration of DRE are shown in Figure 2. Under the established optimal chromatographic conditions, no significant interfering peaks were observed at the analyte elution times, and no interference occurred between IS and the eight analytes.

#### 3.2.2. Linearity and Calibration Curve

All calibration curves exhibited good linearity (*r* ^2^ ≥ 0.9990) over the measured ranges. The calibration curves were linear over the concentrations in the range of 3.75–3749 ng/ml for neoeriocitrin, 1.86–4230 ng/ml for luteolin-7-*O*-*β*-*D*-glucoside, 1.32–1400 ng/ml for astragalin, 1.24–6370 ng/ml for naringin, 0.14–1040 ng/ml for eriodictyol, 2.74–3780 ng/ml for luteolin, 0.12–1210 ng/ml for naringenin, and 5.33–1209 ng/ml for kaempferol, respectively. The linearity regression equation, correlation coefficients, and linear ranges of the eight analytes are shown in Table 2.

#### 3.2.3. Precision and Accuracy

The precision and accuracy of the UPLC-MS/MS method (Table 3) were within acceptable limits. The intraday precision and interday precision (R.S.D.) were within 13.87%, and the accuracy (RE) ranged from −14.79% to −0.25% at three quality control levels.

#### 3.2.4. Recovery and Matrix Effect

The matrix effect and recovery results (Table 4) indicated that no endogenous substances from plasma significantly influenced the ionization of analytes. The matrix effects were within an acceptable range (80.23%–98.99%), and the mean extraction recoveries of the eight analytes and IS were greater than 89.45%.

#### 3.2.5. Stability

The results of stability experiments (Table 5) showed that no significant degradation occurred. The concentrations of the eight analytes measured in the stability study were within −5.66%–3.56%. The data indicated that all analytes in the rat plasma were stable after storage at −20°C for 30 days, after three freeze/thaw cycles, and after storage in the autosampler (4°C) for 36 h.

### 3.3. Pharmacokinetic Studies

**Figure 3:**
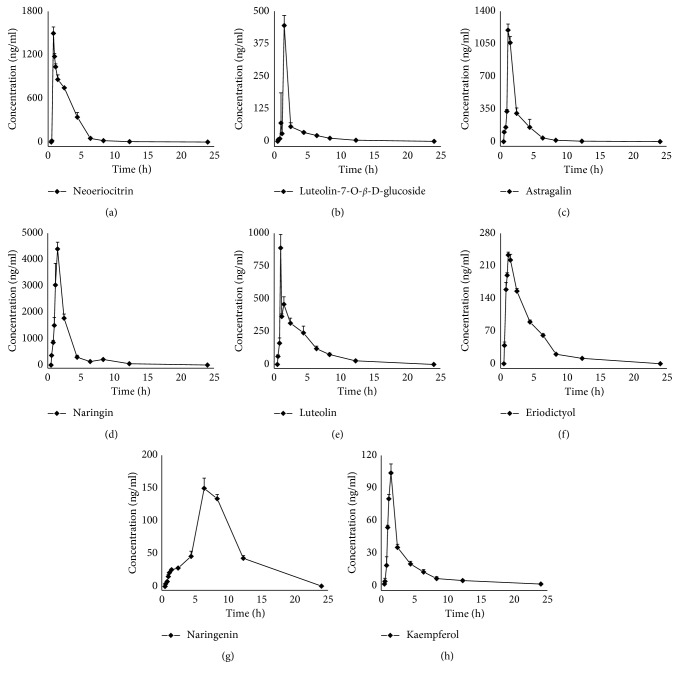
Mean plasma concentration-time curve of eight analytes in rats after oral administration of DRE.

The UPLC-MS/MS method was successfully applied in a pharmacokinetic study of eight flavonoids after oral administration of DRE at a dose of 4 g/kg body weight. Mean plasma concentration-time plots are shown in Figure 3. The major pharmacokinetic parameters are listed in Table 6.

After oral administration to rats, all the analytes were absorbed from the gastrointestinal tract and detected in plasma at 5 min. However, the two highest abundant flavonoids in the DRE, naringin and neoeriocitrin, showed high maximum plasma concentration (*C*_max_) (4414.18 ± 360.38 ng/ml and 1490.98 ± 124.54 ng/ml) and high area under the plasma concentration-time curve from 0 h to infinity AUC (0–∞) (8760.77 ± 347.83 and 3340.34 ± 237.36). Neoeriocitrin still exhibited a shortest *T*_max_ to reach the maximum drug concentration (*T*_max_ = 0.33 h). In the plasma concentration time profile of naringin, another small peak could be seen at 8 h (Figure 3), which is in agreement with the literature where pure naringin was orally administrated to rats [16]. And this phenomenon may be due to the enterohepatic circulation of naringin in rats, which was also reported for other glycosides.

Naringenin, the aglycone of naringin, was absorbed into blood with *T*_max_ at 6 h and eliminated with T_1/2_ at 3.2 h after oral administration of DRE, which was significantly different from the other compounds (*T*_max_ < 2 h). The slow elimination may be due to the fact that orally administrated naringin can be metabolized into naringenin and naringenin glucuronide [17]. Pharmacokinetic characters of naringenin were very close to a previous report [14]. The plasma concentrations of luteolin-7-*O-β*-*D*-glucoside, eriodictyol, and kaempferol were lower, with small area under curve (AUC) and *C*_max_. The *T*_max_ values for luteolin-7-*O-β*-*D*-glucoside and luteolin were 1 h and 0.5 h, respectively, which were in line with a previous report [18, 19]. Luteolin is the metabolite of luteolin-7-*O-β*-*D*-glucoside, the plasma concentration of the former is greater than that of the latter. This may due to the conversion from glycoside to aglucone after oral DRE.

Compared with synthetic drugs, ingredients of plant medicines are complex and have synergistic activity. In this work, we found that most of the analytes reached their maximum concentrations around 2 h after oral administration of DRE. This phenomenon may allow the DRE to exert its greatest pharmacological activity in clinical application.

## 4. Conclusions

In summary, a rapid, sensitive, and selective UPLC-MS/MS method was successfully applied in a pharmacokinetic study for analyzing the active compounds in Chinese herbal medicines. As the main active constituents of DRE, the major pharmacokinetic parameters of eight flavonoids were described for first time. The high efficiency, sensitivity, and selectivity of this multiple components quantitation were guaranteed by UPLC coupled ESI-MS. MRM quantitative analysis mode and two best MRM transitions enhanced the accuracy for each analyte. Results showed that this method could be applied for pharmacokinetic study of the DRE. The present results may provide useful information for better understanding of the absorption of the major constituents of DRE, as well as their potential clinical value.

## Figures and Tables

**Table 1:** Precursor ion and product ion transition and parameters of the analytes used in this study.

Analytes	Retention time (min)	Precursor ion species	MRM transition		Cone (V)	Collision (V)	Dwell time (ms)
			Precursor ion	Product ion			
Neoeriocitrin	2.12	[M + H]^+^	597.5	289.2	25	30	0.2
Luteolin-7-*O-β*-*D*-glucoside	2.33	[M + H]^+^	449.1	287.1	20	20	0.2
Astragalin	2.62	[M + H]^+^	449.1	287.1	30	30	0.2
Naringin	2.73	[M + H]^+^	581.5	273.2	20	25	0.2
Eriodictyol	3.34	[M + H]^+^	289.2	153.1	30	25	0.2
Luteolin	3.63	[M + H]^+^	287.1	153.1	30	30	0.2
Naringenin	4.29	[M + H]^+^	273.2	153.1	20	30	0.2
Kaempferol	4.41	[M + H]^+^	287.1	153.1	30	30	0.2
Quercetin (IS)	3.42	[M + H]^+^	303.2	153.1	20	25	0.2

**Table 2:** Regression data, LLOQ, and LLOD of eight analytes.

Analytes	Calibration curves	Correlation coefficient	Linear range (ng/ml)	LLOQ (ng/ml)	LLOD (ng/ml)
Neoeriocitrin	*y* = 0.132*x* + 11.60	0.9996	3.75–3749	3.75	0.94
Luteolin-7-*O-β*-*D*-glucoside	*y* = 12.48*x* + 339.3	0.9994	1.86–4230	1.86	0.62
Astragalin	*y* = 28.87*x* + 287.0	0.9992	1.32–1400	1.32	0.33
Naringin	*y* = 6.032*x* + 635.9	0.9991	1.24–6370	1.24	0.41
Eriodictyol	*y* = 35.71*x* + 258.9	0.9993	0.14–1040	0.14	0.05
Luteolin	*y* = 12.21*x* + 434.0	0.9990	2.74–3780	2.74	0.91
Naringenin	*y* = 6.269*x* + 128.9	0.9991	0.12–1210	0.12	0.3
Kaempferol	*y* = 7.25x − 79.66	0.9991	5.33–1209	5.33	1.78

**Table 3:** Precision and accuracy for the determination of eight analytes (*n*=6).

Analytes	Concentration spiked (ng/mL)	Intraday			Interday		
		Concentration measured (ng/mL)	Precision (RSD%)	Accuracy (RE%)	Concentration measured (ng/mL)	Precision (RSD%)	Accuracy (RE%)
Neoeriocitrin	40.4	37.31 ± 1.16	3.11	−7.65	36.86 ± 1.28	3.47	−8.76
	202	180.26 ± 10.01	5.55	−13.32	176.01 ± 8.92	5.06	−10.38
	2020	1859.18 ± 76.89	4.14	−7.96	1831.64 ± 65.66	3.58	−9.32
Luteolin-7*-O-β*-*D*-glucoside	5.04	4.12 ± 0.29	7.15	−13.32	4.21 ± 0.38	9.09	−11.44
	50.4	42.55 ± 5.9	13.87	−14.57	45.24 ± 2.69	6.19	−10.23
	504	470.31 ± 13.13	2.79	−6.68	473.18 ± 9.42	1.99	−6.11
Astragalin	5.1	4.12 ± 0.47	11.5	−12.24	4.19 ± 0.4	9.54	−12.94
	102	87.59 ± 8.87	10.13	−14.12	86.62 ± 8.86	10.22	−10.07
	1020	878.88 ± 12.3	1.39	−13.83	840.07 ± 34.9	4.22	−7.64
Naringin	10.2	9.96 ± 0.9	9.01	−2.35	9.53 ± 0.84	8.81	−6.59
	408	400.75 ± 19.57	4.88	−0.8	392.72 ± 12.22	3.09	−2.79
	6120	5989.39 ± 147.05	2.46	−0.37	5935.48 ± 127.9	2.15	−1.27
Eriodictyol	1.02	0.87 ± 0.08	9.46	−13.82	0.87 ± 0.07	8.42	−12.61
	40.8	40.1 ± 4.11	10.25	−0.25	39.63 ± 2.65	6.66	−1.41
	816	728.55 ± 55.67	7.64	−13.26	767.88 ± 35.79	4.74	−8.58
Luteolin	10.1	8.76 ± 0.81	9.29	−13.23	8.69 ± 0.6	6.86	−13.93
	40.4	40.75 ± 3.26	8.00	0.85	39.86 ± 4.24	10.66	−1.34
	1010	925.16 ± 53.96	5.83	−8.39	904.44 ± 65.45	7.26	−10.45
Naringenin	5.2	4.43 ± 0.48	10.72	−14.74	4.33 ± 0.46	10.55	−14.79
	41.6	37.35 ± 2.10	5.63	−10.21	37.93 ± 1.79	4.75	−8.81
	208	181.93 ± 5.97	3.28	−12.53	181.81 ± 8.24	4.54	−12.58
Kaempferol	1.06	0.9 ± 0.08	8.97	−14.25	0.92 ± 0.1	11.06	−13.67
	42.4	37.2 ± 3.06	8.23	−12.26	38.25 ± 2.3	6.06	−9.77
	424	382.89 ± 12.01	3.14	−9.69	376.99 ± 12.36	3.28	−11.08

**Table 4:** Extraction recovery and matrix effect of eight analytes (mean ± SD, *n*=6).

Analytes	Concentration spiked (ng/mL)	Concentration measured (ng/mL)	Extraction recovery (%)		Matrix effect (%)	
			Mean (%)	RSD (%)	Mean (%)	RSD (%)
Neoeriocitrin	40.4	36.64 ± 1.34	90.78 ± 0.99	2.70	90.59 ± 1.69	4.61
	202	173.87 ± 8.38	84.22 ± 7.95	4.54	82.96 ± 8.80	5.10
	2020	1817.87 ± 60.05	91.00 ± 50.84	2.77	88.98 ± 69.26	3.85
Luteolin-7-*O-β*-*D*-glucoside	5.04	4.26 ± 0.43	82.90 ± 0.54	13.03	86.08 ± 0.31	7.08
	50.4	46.59 ± 1.09	91.26 ± 1.47	3.20	93.62 ± 0.71	1.50
	504	474.61 ± 7.57	93.81 ± 6.30	1.33	94.52 ± 8.83	1.85
Astragalin	5.1	4.22 ± 0.36	80.29 ± 0.29	6.99	85.13 ± 0.44	10.13
	102	86.14 ± 8.85	84.96 ± 11.27	13.01	83.94 ± 6.43	7.51
	1020	820.66 ± 46.2	80.23 ± 54.57	6.67	80.68 ± 37.82	4.60
Naringin	10.2	9.31 ± 0.81	94.85 ± 0.75	7.83	87.73 ± 0.86	9.59
	408	388.71 ± 8.55	95.80 ± 7.54	1.93	96.63 ± 9.64	2.47
	6120	5908.53 ± 118.32	98.51 ± 118.35	2.00	98.05 ± 118.30	2.01
Eriodictyol	1.02	0.86 ± 0.07	82.54 ± 0.06	6.87	81.43 ± 0.08	8.93
	40.8	39.4 ± 1.92	98.99 ± 2.27	5.70	97.02 ± 1.57	4.03
	816	787.55 ± 25.85	93.34 ± 27.70	3.53	94.17 ± 24.00	3.03
Luteolin	10.1	8.66 ± 0.49	86.12 ± 0.54	6.20	85.30 ± 0.44	5.09
	40.4	39.41 ± 4.73	97.24 ± 4.65	11.85	97.87 ± 4.80	12.15
	1010	894.08 ± 71.2	89.43 ± 63.90	7.07	87.62 ± 78.49	8.87
Naringenin	5.2	4.27 ± 0.45	81.60 ± 0.52	12.19	82.76 ± 0.38	8.75
	41.6	38.22 ± 1.63	90.02 ± 2.55	6.82	93.74 ± 0.70	1.80
	208	181.76 ± 9.38	85.73 ± 9.89	5.54	89.03 ± 8.86	4.79
Kaempferol	1.06	0.92 ± 0.11	93.71 ± 0.09	9.54	80.50 ± 0.13	14.65
	42.4	38.78 ± 1.93	91.05 ± 2.26	5.84	91.87 ± 1.60	4.10
	424	374.04 ± 12.54	88.47 ± 10.87	2.90	87.96 ± 14.20	3.81

**Table 5:** Stability evaluation results (*n*=6).

Analytes	Spiked (ng/ml)	Autosampler (4°C, 36 h)		Long-term (−20°C, 30 days)		Freeze-thaw (−20°C-room temperature)	
		Measured	RE (%)	Measured	RE (%)	Measured	RE (%)
Neoeriocitrin	40.4	40.29 ± 1.99	−1.00	40.5 ± 2.82	−1.21	39.8 ± 1.92	−2.92
	202	198.79 ± 11.22	2.58	206.55 ± 12.4	1.72	205.16 ± 23.24	1.36
	2020	2001.91 ± 21.3	−0.90	2012.25 ± 27.81	−0.37	2032.19 ± 45.28	0.61
Luteolin-7-*O-β*-*D*-glucoside	5.04	5.02 ± 0.18	−3.12	4.99 ± 0.07	−2.34	5.01 ± 0.21	−1.26
	50.4	49.44 ± 1.55	−1.38	49.37 ± 3.07	−3.64	49.55 ± 8.55	−2.45
	504	494.39 ± 10.55	−1.90	493.07 ± 13.07	−2.16	491.55 ± 18.55	−2.47
Astragalin	5.1	5.02 ± 0.18	−1.63	4.99 ± 0.07	−2.18	5.01 ± 0.21	−1.79
	102	101.10 ± 4.29	−2.79	99.7 ± 3.76	−4.13	102.04 ± 4	−1.88
	1020	1006.41 ± 30.28	3.56	1002.46 ± 35.15	1.22	1000.65 ± 34.59	1.04
Naringin	10.2	10.5 ± 0.14	−4.02	10.3 ± 0.09	−0.27	9.9 ± 0.18	−1.11
	408	401.01 ± 8.13	−0.24	400.90 ± 9.65	−0.51	403.52 ± 11.42	−0.11
	6120	6008.15 ± 38.45	−5.66	6010.28 ± 22.59	−3.22	6009.12 ± 43.76	−4.99
Eriodictyol	1.02	0.96 ± 0.06	−1.30	1.06 ± 0.06	−4.22	0.99 ± 0.07	−2.84
	40.8	38.83 ± 2.5	−0.44	39.87 ± 1.83	2.28	39.00 ± 2.32	−2.64
	816	834.09 ± 23.38	−0.70	837.06 ± 26.59	−0.34	824.21 ± 18.77	−1.88
Luteolin	10.1	10.2 ± 0.04	3.54	9.97 ± 0.09	−0.13	9.96 ± 0.1	−1.04
	40.4	37.85 ± 1.42	0.94	38.08 ± 1.5	1.55	39.39 ± 1.9	−0.29
	1010	1000.91 ± 21.3	−1.91	1006.25 ± 27.81	−0.25	1016.19 ± 45.28	0.64
Naringenin	5.2	5.1 ± 0.06	0.92	5.1 ± 0.11	0.89	5.08 ± 0.11	0.66
	41.6	41.50 ± 3.05	−1.20	41.33 ± 3.09	−1.60	41.13 ± 3.11	−2.06
	208	207.10 ± 4.29	−4.79	208.70 ± 5.76	−8.13	204.04 ± 8	−3.76
Kaempferol	1.06	1.05 ± 0.14	−3.02	1.03 ± 0.09	−0.49	1.09 ± 0.18	−1.71
	42.4	40.59 ± 1.39	−1.07	41.15 ± 2.32	−1.34	41.8 ± 1.58	−2.67
	424	419.01 ± 7.42	−0.44	427.9 ± 3.74	−0.46	419.52 ± 8.323	−0.31

**Table 6:** Pharmacokinetic parameters of eight analytes in rat plasma after oral administration of DRE (mean ± SD, *n*=6).

Analytes	AUC (0–*t*) (ng·h/ml)	AUC (0–*∞*) (ng·h/ml)	*T*_max_ (h)	T_1/2_ (h)	*C*_max_ (ng/kg)	MRT (0–*t*) (h)
Neoeriocitrin	3327.71 ± 238.61	3340.34 ± 237.36	0.33	1.69 ± 0.28	1490.98 ± 124.54	2.16 ± 0.15
Luteolin-7-*O-β*-*D*-glucoside	550.97 ± 44.68	565.98 ± 56.06	1	2.34 ± 0.61	440.37 ± 52.16	2.55 ± 0.28
Astragalin	1999.53 ± 338.21	2012.24 ± 331.76	0.67	1.94 ± 0.6	1203.63 ± 90.89	1.97 ± 0.25
Naringin	8669.19 ± 321.61	8760.77 ± 347.83	1	2.51 ± 0.94	4414.18 ± 360.38	2.36 ± 0.08
Eriodictyol	894.09 ± 33.5	929.83 ± 26.85	0.67	2.49 ± 0.19	232.76 ± 8.52	3.24 ± 0.14
Luteolin	2040.99 ± 219.28	2144.07 ± 173.66	1	2.62 ± 0.64	895.98 ± 140.34	3.51 ± 0.11
Naringenin	946.98 ± 49.06	1164.21 ± 58.65	6	3.21 ± 0.54	153.87 ± 17.39	6.89 ± 0.04
Kaempferol	232.81 ± 29.32	244.43 ± 34.91	1	2.86 ± 0.58	102.82 ± 11.61	2.84 ± 0.32

## Data Availability

The data (Figures 1–3) used to support the findings of this study are included within the article and the supplementary information files.

## Abbreviations

UPLC-MS/MS: Ultraperformance liquid chromatography tandem mass spectrometry
DRE: *Drynariae rhizoma* extract
MRM: Multiple reaction monitoring
IS: Internal standard
ESI+: Positive ion mode of electrospray ionization
ACN: Acetonitrile
MeOH: Methanol
QC: Quality control
LLOD: Lower limit of detection
LLOQ: Lower limit of quantification
S/N: Signal-noise ratio
RSD: Relative standard deviations
RE: Relative error.

## Supplementary Materials

Supplementary Materials An experimental procedure is described in the graphic abstract. The experiment was divided into drug extraction, rats' intragastric administration, plasma sample treatment, UPLC-MS/MS analysis, and data processing.
