# Supplementary figures and images for: Pharmacokinetics of Eight Flavonoids in Rats Assayed by UPLC-MS/MS after Oral Administration of Drynariae rhizoma Extract
Source: J Anal Methods Chem. 2018 Dec 18;2018:4789196. doi: 10.1155/2018/4789196 (PMC6312611; doi:10.1155/2018/4789196)

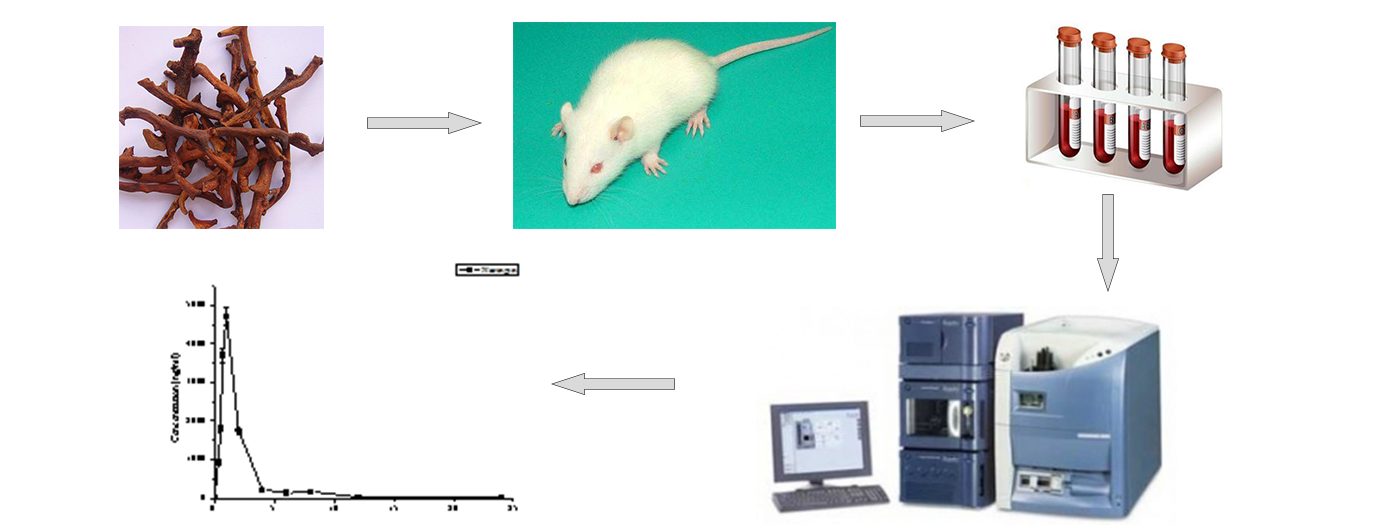

Supplement: Supplementary Materials — An experimental procedure is described in the graphic abstract. The experiment was divided into drug extraction, rats' intragastric administration, plasma sample treatment, UPLC-MS/MS analysis, and data processing. [file 4789196.f1.tif]
